# Association of Diabetes Mellitus and Alcohol Abuse with Cancer: Molecular Mechanisms and Clinical Significance

**DOI:** 10.3390/cells10113077

**Published:** 2021-11-08

**Authors:** Bao Q. Lam, Rashmi Srivastava, Jason Morvant, Sharmila Shankar, Rakesh K. Srivastava

**Affiliations:** 1 Stanley S. Scott Cancer Center, Louisiana State University Health Sciences Center, New Orleans, LA 70112, USA; lamquocbao7419@gmail.com (B.Q.L.); sshankar9@gmail.com (S.S.); 2 Department of Pharmacology, Louisiana State University Health Sciences Center, New Orleans, LA 70112, USA; rsssrs12@gmail.com; 3 Department of Surgery, Ochsner Health System, 120 Ochsner Boulevard, Gretna, LA 70056, USA; jasonmorvant@gmail.com; 4 A.B. Freeman School of Business, Tulane University, New Orleans, LA 70118, USA; 5 Department of Genetics, Louisiana State University Health Sciences Center, New Orleans, LA 70112, USA; 6 John W. Deming Department of Medicine, School of Medicine, Tulane University, New Orleans, LA 70112, USA; 7 Southeast Louisiana Veterans Health Care System, New Orleans, LA 70119, USA

**Keywords:** diabetes mellitus, alcoholism, breast cancer, pancreatic cancers, gastric cancer, colorectal cancer, hepatocellular carcinoma, bladder cancer

## Abstract

Diabetes mellitus (DM), one of the metabolic diseases which is characterized by sustained hyperglycemia, is a life-threatening disease. The global prevalence of DM is on the rise, mainly in low- and middle-income countries. Diabetes is a major cause of blindness, heart attacks, kidney failure, stroke, and lower limb amputation. Type 2 diabetes mellitus (T2DM) is a form of diabetes that is characterized by high blood sugar and insulin resistance. T2DM can be prevented or delayed by a healthy diet, regular physical activity, maintaining normal body weight, and avoiding alcohol and tobacco use. Ethanol and its metabolites can cause differentiation defects in stem cells and promote inflammatory injury and carcinogenesis in several tissues. Recent studies have suggested that diabetes can be treated, and its consequences can be avoided or delayed with proper management. DM has a greater risk for several cancers, such as breast, colorectal, endometrial, pancreatic, gallbladder, renal, and liver cancer. The incidence of cancer is significantly higher in patients with DM than in those without DM. In addition to DM, alcohol abuse is also a risk factor for many cancers. We present a review of the recent studies investigating the association of both DM and alcohol abuse with cancer incidence.

## 1. Introduction

The possible biological links between diabetes mellitus or impaired glucose tolerance and cancer comprise hyperinsulinemia, hyperglycemia, and fat-induced chronic inflammation. DM is a known risk factor for several cancers [1], resulting from insulin resistance induced by a paraneoplastic syndrome [2] or pancreatic β-cell dysfunction [3]. Mechanistically, hyperglycemia may cause hyperinsulinemia, providing growth signals to positively stimulate the expansion of cancer [4,5,6]. In addition, it has been demonstrated that moderate alcohol intake had no significant impact, whereas high alcohol intake was associated with an increased risk of breast and gastrointestinal cancer [7,8,9,10].

According to the National Diabetes Statistics Report, a periodical publication by the Centers for Disease Control and Prevention (CDC), during 1999–2016, the age-adjusted prevalence of total diabetes significantly increased among adults aged 18 years or older. Prevalence estimates were 9.5% in 1999–2002 and 12.0% in 2013–2016. Among the overall US population, the crude estimates for 2018 were that 34.2 million people of all ages or 10.5% of the US population had diabetes. Furthermore, 34.1 million adults aged 18 years or older, or 13.0% of all US adults, had diabetes. Age-adjusted data for 2017–2018 indicated that non-Hispanic blacks (8.2 per 1000 persons) and people of Hispanic origin (9.7 per 1000 persons) had a higher incidence of diabetes compared to non-Hispanic whites (5.0 per 1000 persons). According to the National Institute of Diabetes and Digestive and Kidney Diseases, diabetes is the seventh leading cause of death in the United States.

**Figure 1:**
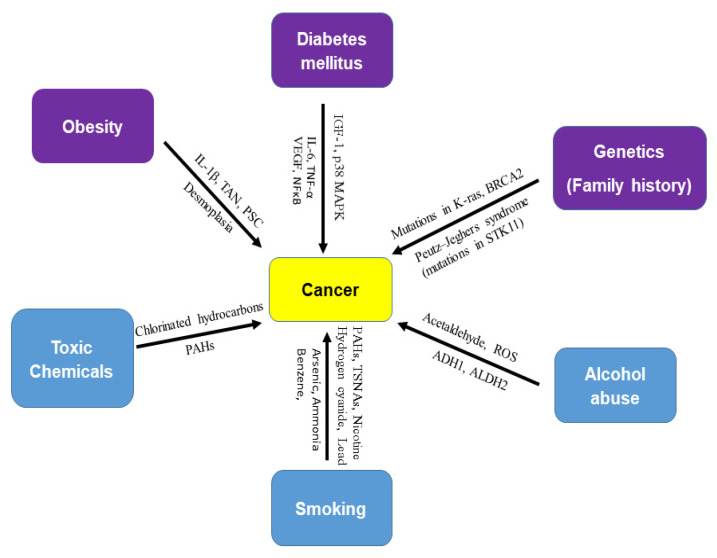
Risk factors of cancer. There are several risk factors for cancer. Obesity (IL-1β, TAN, PSC, desmoplasia), diabetes mellitus (IGF-1, p38 MAPK, IL-6, TNF-β, VEGF, and NF-κB), and genetics (mutations in K-ras, BRCA2, and STK11) are biological risk factors for cancer. Toxic chemicals (chlorinated hydrocarbons and polycyclic aromatic hydrocarbons), alcohol abuse (acetaldehyde, ROS, ADH1, and ALDH2), and smoking (nicotine, hydrogen cyanide, formaldehyde, lead, arsenic, ammonia, benzene, carbon monoxide, nitrosamines, and polycyclic aromatic hydrocarbons), are external or environmental risk factors of cancer. Smoking is known as a strong carcinogen in many cancers. Most cancer cases are attributed to environmental factors but a small percentage are involved in gene mutations and hereditary traces. Peutz–Jeghers syndrome (PJS) is caused by mutations in the tumor suppressor STK11 gene.

In 2017, the International Agency for Research on Cancer (IARC) concluded that obesity is a risk factor of cancer of 13 anatomic sites [11]. The direct association of diabetes mellitus with pancreatic, liver, breast, endometrium, bladder, and kidney cancer has been demonstrated. In addition to obesity and diabetes, other risk factors of cancer are alcohol abuse, genetics (family history), smoking, and exposure to toxic chemicals (Figure 1). Recent studies have shown an association between the incidence of cancer and anti-diabetic medications. Furthermore, the use of metformin (a drug for type 2 diabetes mellitus) is associated with a reduced risk of cancer [12,13,14,15,16] or cancer mortality [17]. The objective of this review paper is to update and summarize the mechanisms of association of diabetes mellitus and alcohol abuse with major cancer.

## 2. Diabetes

Diabetes is a chronic disease that occurs either when the pancreas does not produce enough insulin (a hormone that regulates blood sugar) or when the body cannot effectively use the insulin it produces. There are three major types of diabetes mellitus (DM)—type 1, type 2, and gestational diabetes. Type 1 diabetes mellitus (T1DM), insulin-dependent diabetes mellitus, or juvenile-onset diabetes may account for about 5% of all cases of diabetes. T1DM is characterized by a genetic predisposition manifested in one of several human leukocyte antigens. Type 2 diabetes mellitus (T2DM)**,** non-insulin-dependent diabetes mellitus, or adult-onset diabetes, account for about 90% (285 million people), and this number is projected to grow to 438 million by 2030. T2DM is a form of diabetes mainly characterized by high blood glucose, insulin resistance, and relatively a weaker insulin-stimulated response under hyperglycemic conditions. In obese individuals with euglycemia, peripheral insulin resistance is present but compensated by increased insulin secretion [18,19,20]. Insulin resistance progressively worsens in predisposed individuals, along with progressive β-cell dysfunction and the reduction of β-cell mass, eventually leading to T2DM [18,19,20]. DM is not only related to retinopathy, neuropathy, nephropathy, and cardiovascular diseases but is also related to several liver diseases such as nonalcoholic fatty liver disease (NAFLD), steatohepatitis, and liver cirrhosis. Long-standing T2DM, insulin resistance, and obesity have been shown to modestly increase the risk of several types of cancers [21,22,23,24,25,26,27,28]. According to the World Health Organization, obesity is defined by the body mass index of more than 30 kg/m^2^, and overweight is 25–30 kg/m^2^. Obesity causes chronic inflammation of the body and is a risk factor for many cancers. There are a number of conditions associated with diabetes, such as thyroid disease, coeliac disease, polycystic ovary syndrome, diabetes insipidus, necrobiosis lipoidica diabeticorum, mastopathy, muscular conditions, dental health complications, and certain types of cancer. Growing evidence suggests that patients with colorectal, breast, liver, endometrial, and gastric cancers and leukemia [29,30] who also have DM are at increased risk of cancer recurrence and mortality [31].

**Figure 2:**
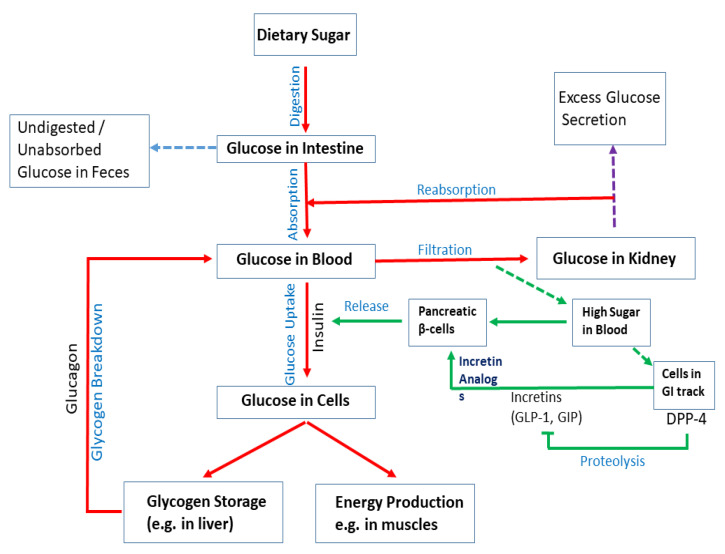
Mechanisms of diabetes. Upon digestion of dietary sugar, glucose is absorbed by the intestine, which causes an increase in the blood glucose level. Glucose levels increase in the blood and insulin is secreted by pancreatic β-cells. Insulin enhances uptake of glucose into cells. Glucose is stored as glycogen in the liver or utilized for energy production in muscles. If blood sugar levels are low, glucagon breaks down glycogen in the liver to release glucose and increase glucose levels. Intestinal cells secrete DPP4, which inhibits the production of incretins (e.g., GLP-1 and GIP). They act on pancreatic β-cells to regulate insulin production. Although glucagon induces hepatic glucose production, insulin acts as a potent inhibitor of glucose production when its concentration in the blood rises. Undigested/unabsorbed glucose is excreted from the body.

Upon digestion of dietary sugar, glucose is absorbed by the intestine, which results in a rise in the blood glucose level (Figure 2). In the liver, insulin regulates glucose production/utilization. When glucose levels increase in the blood and insulin is secreted by pancreatic β-cells. Intestinal cells secrete DPP4, which inhibits the production of incretins such as GLP-1 and GIP; they act on pancreatic β-cells to regulate insulin production. In physiological states, the combined action of glucagon and insulin allows the precise regulation of hepatic glucose output. Although glucagon induces hepatic glucose production, insulin acts as a potent inhibitor of glucose production when its concentration in the blood rises. In addition to inducing glycogen synthesis, insulin also inhibits hepatic glucose production. In the case of insulin resistance, physiologic levels of circulating insulin are insufficient to elicit the appropriate insulin response in hepatic cells. In the liver, insulin resistance impairs glycogen synthesis, fails to suppress glucose production, enhances lipogenesis, and increases the synthesis of proteins. Insulin resistance occurs due to a decrease in the metabolic response of insulin-responsive cells to insulin or, at a systemic level, an impaired/lower response to circulating insulin by blood glucose levels. In skeletal muscle, insulin resistance is considered to be an important extra-pancreatic factor in the development of T2DM. Under physiological conditions, insulin stimulates muscle glycogen synthesis by enhancing glucose uptake from plasma.

## 3. Alcohol Metabolisms

**Figure 3:**
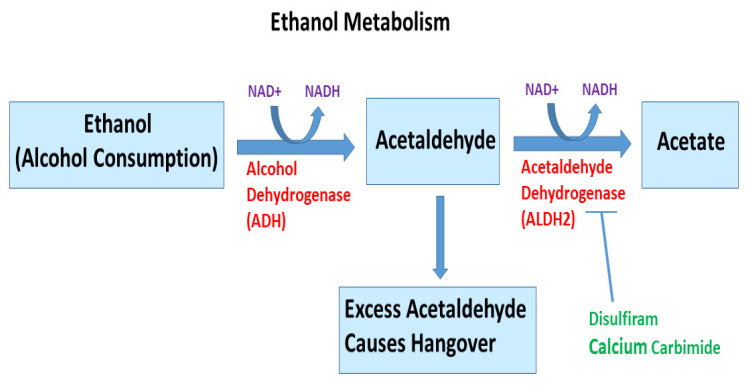
Ethanol metabolism. Ethanol, an alcohol found in nature and alcoholic drinks, is metabolized through a complex catabolic metabolic pathway. In humans, in the presence of enzyme alcohol dehydrogenase and NAD^+^, ethanol is metabolized to acetaldehyde, which is further converted to acetate and acetyl-CoA in the presence of acetaldehyde dehydrogenase and NAD^+^. Once acetyl-CoA is formed, it becomes a substrate for the citric acid cycle, ultimately producing cellular energy and releasing water and carbon dioxide (not shown). The liver is the major organ that metabolizes ethanol. Disulfiram and calcium carbamide can inhibit ALDH2 activity. Excess acetaldehyde can cause hangover.

Alcohol has been classified as a human carcinogen for the liver by the International Agency for Research on Cancer (IARC). Ethanol metabolism is shown in Figure 3. Chronic ingestion of alcohol and its metabolite acetaldehyde may initiate and/or promote the development of cancer in the liver, oral cavity, esophagus, stomach, gastrointestinal tract, pancreas, prostate, and female breast. During ethanol metabolism, ethanol is oxidized to acetaldehyde by alcohol dehydrogenases (ADH) in the presence of NAD^+^ [32,33]. Another source of acetaldehyde is bacteria living in the gastrointestinal tract [34,35]. The CYP p450 (CYP2E1) pathway also metabolizes ethanol into acetaldehyde, as well as reactive oxygen species (ROS). However, this pathway is more active when a high alcohol intake is consumed [36]. Acetaldehyde is further metabolized into acetate by aldehyde dehydrogenase (ALDH), in particular, the form encoded by ALDH2 on chromosome 12 [36]. The breakdown of alcohol into acetaldehyde and then acetate mainly takes place in the liver. Some alcohol metabolism also occurs in other tissues, such as the pancreas and the brain. Additionally, small amounts of alcohol are metabolized to acetaldehyde in the gastrointestinal tract. Recently, ALDH2 deficiency has been linked with the risk, pathogenesis, and prognosis of various cancers, and has emerged as a promising therapeutic target [37]. Ethanol and acetaldehyde can cause differentiation defects in stem cells and promote inflammatory injury and carcinogenesis in several tissues [38,39]. Disulfiram and calcium carbamide can inhibit ALDH2 activity.

Alcohol (ethanol) and its major metabolite, acetaldehyde, are classified by the IARC as Group 1 carcinogens [40]. Although alcohol has been classified as carcinogenic, it is thought that metabolites of ethanol are probably the most important in terms of causal carcinogens. This is based mainly on the observation that metabolites of ethanol such as acetaldehyde and ROS can induce DNA lesions [41]. Several factors have been shown to contribute to the development of alcohol-associated cancer [42,43,44,45,46]. Recent studies have demonstrated that the effect of alcohol is modulated by polymorphisms in genes encoding enzymes for ethanol metabolism (e.g., ADH, ALDH, and cytochrome P450 2E1), folate metabolism, and DNA repair. The mechanisms by which alcohol consumption exerts its carcinogenic effect are not well understood. Actions such as a genotoxic effect of acetaldehyde, increased estrogen concentrations, a role as a solvent for tobacco carcinogens, production of ROS and nitrogen species, and changes in folate metabolism have been implicated. Acetaldehyde reacts with DNA and acts as a carcinogen. In addition, highly reactive, oxygen-containing molecules (generated during alcohol metabolism) can damage the DNA and induce carcinogenesis [47,48]. In healthy adults, the spontaneous consumption of alcoholic beverages within 30 g ethanol per day for men and 15 g per day for women is considered acceptable. The daily consumption of more than 80 g of alcohol (more than five to six drinks) with smoking increases the risk of developing cancers by 50 fold or more [34,49].

## 4. Diabetes, Alcohol, and Breast Cancer

Obesity, diabetes mellitus, and unhealthy lifestyle behaviors (alcohol dependence, smoking, low physical activity) are risk factors for breast cancer [50]. The Mediterranean diet, which consists of fish, monounsaturated fats from olive oil, a high omega-3-to-omega-6 ratio, fruits, vegetables, whole grains, legumes/nuts, and moderate alcohol consumption, has been shown to reduce the risk and prevent the development of depression, diabetes, obesity, and breast cancer [51,52]. Obese or overweight women at the time of breast cancer diagnosis are at increased risk of cancer recurrence and mortality compared with leaner women [53,54]. Obesity is associated with adverse outcomes in both pre- and postmenopausal women with breast cancer [53]. Hyperinsulinemia may promote mammary carcinogenesis. Insulin resistance has been linked to an increased risk of breast cancer and is also characteristic of T2DM [53]. The Nurses’ Health Study showed that women with T2DM had a modestly elevated incidence of breast cancer (hazard ratio (HR) = 1.17; 95% CI 1.01–1.35) compared with women without diabetes [54]. Metformin compared to the use of other oral antidiabetic drugs is associated with a lower risk of cancer in patients with T2DM [55]. Metformin may induce ferroptosis by inhibiting autophagy via lncRNA-H19 [56]. Metformin induced the activation of AMP-induced protein kinase (AMPK), suppressed the phosphorylated-eukaryotic translation initiation factor 4E-binding protein 1 (p-4E-BP1), decreased cyclin D1 levels, and inhibited COX-2 expression [57]. Metformin inhibits mTORC1 directly or through the inhibition of AMPK. The inhibition of AMPK by metformin results in the induction of p53 and the inhibition of Mdm2. Interestingly, metformin can also downregulate cyclin D1 and upregulate p53 expression through an AMPKα-independent mechanism in breast cancer [58]. Furthermore, metformin suppresses tumor growth by inhibiting the activation of Akt, MEK/ERK, NFkB, and Stat3.

Drinking within recommended limits is socially acceptable and prevalent in most of the world. It is believed that consuming no more than one alcoholic drink per day, preferably red wine, is beneficial for health. Strong epidemiological data exist showing associations between moderate alcohol consumption and the risk of diabetes and breast cancer. Alcohol intake has been associated with an increased risk of breast cancer and decreased risk of coronary heart disease [59]. CYP2E1 exerts a significant role in mammary carcinogenesis, and thus provides a potential link between ethanol metabolism and advanced stages of breast cancer [60]. Factors that are differently distributed among ethnic groups, such as obesity, diabetes, metabolic syndrome, alcohol consumption, and smoking, predict survival after breast cancer diagnosis and therefore might mediate part of the observed social disparity in survival [61].

## 5. Diabetes, Alcohol, and Pancreatic Cancer

Pancreatic cancer (PC) is one of the ten most common cancers in humans. Most of these cases are pancreatic exocrine cancer; only 1–2% of cases of PC are neuroendocrine tumors. It causes 7% of all cancer deaths. It is the fourth highest cause of cancer-related death in both men and women in the United States each year [62]. In the United States, the number of new cases of PC was 12.4 per 100,000 men and women per year based on 2009–2013 cases. The five-year survival rate for PC is 7.7% (2006–2012) [63]. The common risk factors for developing PC are tobacco products, obesity, diabetes, chronic pancreatitis, hereditary conditions, and a family history of PC [62].

DM, or impaired glucose tolerance, is concurrently present in 50–80% of patients with PC. DM is a known risk factor for PC [64,65,66], and in another aspect, new-onset DM could be an early manifestation of PC [1], resulting from insulin resistance induced by a paraneoplastic syndrome [2] or pancreatic β-cell dysfunction [3]. In addition, it has been demonstrated that moderate alcohol intake had no significant impact, whereas high alcohol intake was associated with an increased risk of PC [7,8,9].

There was a three-fold risk of pancreatic malignancy in patients with diabetes [67]. Consistently, the risk of PC increased with a longer duration of diabetes in a prospective study with a hazard risk of 2.0 in both men and women [68,69]. In a Taiwanese cohort, diabetes mellitus was associated with a relative risk of 2.75 in relation to developing PC and other gastrointestinal tumors [70]. It was established that HbA1C is a predictor and prognostic factor in PC [71], requiring further studies to clarify the function of HbA1C in the pathogenesis of PC. Under-treated DM patients had a higher risk for PC than all DM populations [72]. New-onset DM is significantly associated with reduced survival, whereas long-standing DM does not affect overall survival significantly [73]. The new-onset DM could be attributed to the paraneoplastic phenomenon mediated by tumor-secreted products, whereas long-standing DM is induced by non-paraneoplastic factors.

Several studies show that obesity is one of the leading risk factors for PC [74,75,76]. Along with an increased risk of developing PC, patients with increased pancreatic fat have poorer outcomes than those who develop cancer in a lean pancreas [77]. Emerging evidence shows that the increase of certain hormones in obese patients, such as insulin, adipokines, and resistine and systemic oxidative stress may have a role in the development of PC [78,79].

Adipocytes in obesity secrete high IL-1β recruit tumor-associated neutrophils (TAN), which induce the activation of pancreatic stellate cells (PSC). IL-1β, TAN, and PSC induce the aggravation of desmoplasia, which is modulated by the angiotensin-II type-1 receptor and promotes PC progression [80]. Obesity is associated with increased systemic levels of placental growth factor (PLGF) [81,82]. PLGF and its receptor VEGFR-1 have been shown to modulate tumor angiogenesis and promote tumor-associated macrophage (TAM) recruitment and activity [83,84,85]. Targeting PLGF/VEGFR-1 signaling reprogramed the tumor immune microenvironment and inhibited obesity-induced PC progression [86].

The markedly higher risk of PC in patients with new-onset diabetes when compared with long-standing diabetes indicates that PC itself can cause diabetes. Based on epidemiological studies, most PC patients have glucose intolerance [87] and about 80% of PC patients are either glycemic or diabetic. Dysfunctions of β-cell and insulin resistance are often seen in PC. Serum betatrophin levels were significantly correlated with PC-associated diabetes [88]. Betatrophin has been implicated in glucose metabolism and β-cell proliferation [89]. It promotes pancreatic β-cell proliferation and improves glucose intolerance in mouse models of insulin resistance [90]. Typical risk factors for T2DM (such as older age, obesity, and a family history of diabetes) are also risk factors for pancreatic cancer-induced diabetes [91]. Hence, it is difficult to identify if diabetes is the cause or outcome of PC. However, in some situations, pancreatic cancer-induced diabetes has also been reported. Resection of the cancerous parts of the pancreas improves diabetes [91,92]. Furthermore, supernatants from PC cell lines have been shown to induce insulin resistance in cultured hepatocytes [93,94] and myoblasts [95], as well as β-cell dysfunction in vivo [96] and in vitro [97,98,99,100,101]. Gene profiling using microarray analysis of PC cell lines showed 18 upregulated proteins [98], among which adrenomedullin, a 52-amino-acid peptide known to inhibit insulin secretion [102,103], was identified. Another mechanism of PC-induced diabetes is insulin resistance. Similarly to T2DM, this resistance in PC is thought to occur at the post-receptor level. Differences in glycogen synthesis and glycogen breakdown in skeletal muscles were observed in patients with PC-induced diabetes compared with those with non-diabetes PC and healthy controls. Furthermore, islet amyloid polypeptide (IAPP) [104] and S-100A8 N-terminal peptide [105,106] also induce insulin resistance in vitro.

Heavy alcohol intake has been associated with a higher risk of pancreatitis. The risk of developing pancreatitis increases with increasing doses of alcohol (80 to 150 g/d). In comparison to pancreatitis, the role of alcohol consumption remains less clear in PC. Low to moderate alcohol consumption does not appear to be associated with PC risk, and only chronic heavy drinking increases the risk of PC. If the patients consume alcohol, the acetaldehyde concentrations in the stomach increase 6.5-fold [107].

It is also possible that heavy alcohol consumption influences PC development independently of chronic pancreatitis. A positive association between alcohol intake and PC risk was observed only among individuals with low total folate intake; the association was attenuated among individuals with high total folate intake [108]. Alcohol has been demonstrated to regulate folate bioavailability and interrupt critical folate-driven biological processes; inadequate levels of folate can disrupt DNA methylation, synthesis, and repair [109]. In addition, it is plausible that alcohol consumption may only affect PC risk among individuals with low folate intake [109]. Individuals with heavy consumption of alcohol may have a reduced folate status, making the pancreas susceptible to carcinogenesis [110,111]. However, in another study, no evidence for the interaction of alcohol consumption with folate in PC risk was found [112]. This disparity could be due to the low number of cases in this study.

## 6. Diabetes, Alcohol, and Liver Cancer

The major risk factors for hepatocellular carcinoma (HCC), the most frequent histological type of primary liver cancer, are persistent infection with hepatitis B virus (HBV) and hepatitis C virus (HCV), both of which increase the risk of liver cancer to 20-fold [113]. Other established risk factors include non-alcoholic fatty liver disease (NAFLD), tobacco smoking, alcohol abuse, exposure to aflatoxin-contaminated food, and some rare inherited disorders, including hereditary hemochromatosis [47,48,114,115,116,117].

**Figure 4:**
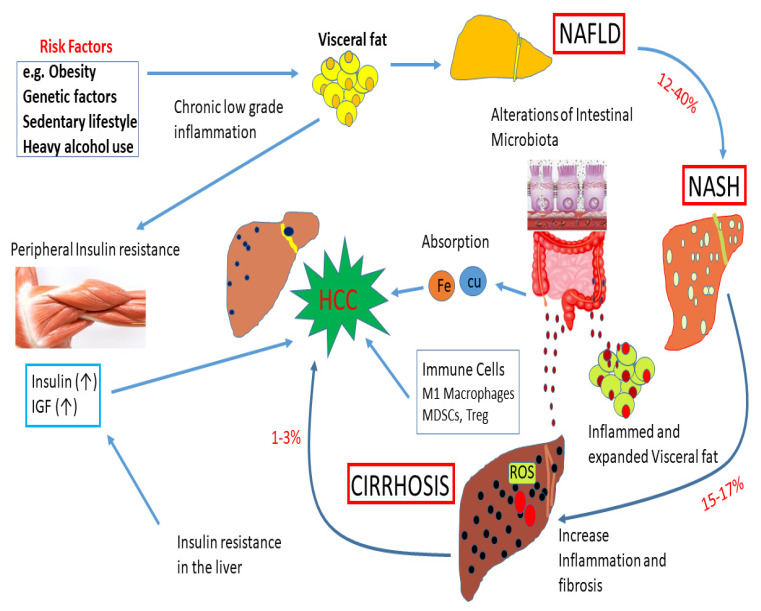
Impact of obesity, genetic factors, and a sedentary lifestyle on liver diseases. Obesity, genetic factors, and a sedentary lifestyle enhance the deposition of visceral fat. These events can play a significant role in the development of non-alcoholic fatty liver disease (NAFLD). About 12–40% of cases of NAFLD develop into NASH. About 15–17% of NASH cases may advance to cirrhosis due to an increase in inflammation and fibrosis. A subset of cirrhosis (1–3%) will finally advance to hepatocellular carcinoma (HCC). High levels of insulin and IGF-1 during insulin resistance can accelerate the development of HCC. Alterations of intestinal microbiota can cause inflammation and visceral fat deposition. The absorption of Fe and Cu in the gut, and immune cells, M1 macrophages, myeloid-derived suppressor cells (MDSCs), and Treg can regulate the development of HCC. In addition, ROS and fatty acid ethyl esters induce cellular damage.

Emerging evidence supports a positive association between diabetes and liver cancer [118,119]. The impact of obesity, genetic factors, and a sedentary lifestyle on liver diseases is shown in Figure 4. Diabetic patients undergoing insulin treatment are at increased risk of developing HCC because of the mitogenic effects of high insulin [120]. Patients with diabetes are also more likely to have hepatic steatosis [121,122], and a fatty liver may increase liver cancer risk through excess inflammation, oxidative stress, and other mechanisms [123,124,125,126]. Steatosis can lead to nonalcoholic steatohepatitis and even to fibrosis, cancer, and cirrhosis of the liver. Serum glucose was found to be positively associated with liver cancer (OR 1.88); serum insulin and diabetes were associated with a higher risk of liver cancer mortality (OR 3.42 and 2.95, respectively) [127]. Insulin can exert a potentially mitogenic effect by activating the insulin receptor and then triggering intracellular signaling cascades that have the potential to be both mitogenic and anti-apoptotic due to activation of the phosphatidylinositol 3-kinase-AKT pathway [128] and by interacting with the insulin-like growth factor-1 (IGF-1) receptors which enhance cancer cell proliferation [129]. Elevated insulin can also increase free IGF-1 (i.e., the bioactive form of IGF-1) in the blood by reducing the production of IGF-1-binding proteins 1 and 2 in the liver, thereby positively enhancing tumor development [130]. NAFLD development and liver insulin resistance are linked through an interaction between the accumulation of free fatty acids, hepatic inflammation, and increased oxidative stress. Hyperglycemia among diabetic patients can increase oxidative stress in the cells due to an overload of glucose oxidation and other mechanisms, leading to the production of ROS such as hydroxyl radical [131]. ROS can bind DNA, can cause gene mutations, and may induce cancer development. Obesity in DM promotes elevated levels of pro-inflammatory factors such as tumor necrosis factor-alpha (TNF-α) and interleukin-6 and decreases the levels of adiponectin with anti-inflammatory actions, resulting in chronic inflammation, which can promote hepatocarcinogenesis [132].

In addition to the carcinogenicity of acetaldehyde, several other biological mechanisms have been proposed to explain the effect of alcohol on HCC. These include chronic inflammation, resulting in increased oxidative stress and the induction of cytochrome P-450 2E1, leading to increased ROS production, lipid peroxidation, DNA damage, a decrease in antioxidant defense and DNA repair, disturbed methyl transfer associated with DNA hypomethylation, decreased hepatic retinoic acid, iron overload, and impairment of the immune system [133]. ALDH2 deficiency exacerbates alcohol-associated HCC development. Furthermore, after chronic alcohol exposure, ALDH2-deficient hepatocytes produced a large amount of harmful oxidized mitochondrial DNA via extracellular vesicles, which are capable of activating multiple oncogenic pathways and promoting HCC development [37].

## 7. Diabetes, Alcohol, and Gastric Cancer

The morbidity and mortality rate of gastric cancer has declined rapidly over the past few decades, probably due to the recognition of certain risk factors such as *Helicobacter pylori* and dietary and environmental risks factors [134]. Gastric cancer is more common in men and people aged 50 years or older. Obesity, smoking, and *Helicobacter pylori* (*H. pylori*) infection are important risk factors [113,135]. Few studies have focused on the relationship between DM and the development of gastric cancer. Some systematic meta-analysis reviews have shown the higher risk and mortality of gastric cancer in DM patients [136,137]. However, others have shown that there is no clear association between DM and the risk of gastric cancer [138,139]. Recently, it was found that DM increases the risk of early gastric cancer development within an average of 70 months of follow-up [140]. All gastric cancers in this study were identified in patients with gastric atrophy, and no cancer was identified in patients without gastric atrophy, which is a typical presentation of *H. pylori* infection. However, a small percentage of gastric atrophy patients will develop gastric cancer. It was also reported that hyperglycemia and HbA1c increased the risk of gastric cancer induced by *H. pylori* infection [141]. The production of ROS, which causes DNA damage [142], is increased in hyperglycemia, and a high glucose level itself has been shown to contribute to DNA damage not only in vitro but also in patients with DM [143]. Hyperinsulin in DM has a mitogenic effect by activating the mitogen-activated protein (MAP) or phosphoinositide 3 (PI3) kinase pathway via insulin receptors [144,145] and the signaling of insulin-like growth factor receptors (IGF-Rs) [146].

The relationship between drinking alcohol and the risk of gastric cancer is biologically plausible. However, it is still a matter of debate whether alcohol consumption elevates the risk of gastric cancer. Previously, moderate and heavy drinking increased the risk of gastric cancer [147]. However, in another study, no association of gastric cancer with dose-dependent alcohol consumption was observed [148]. Instead, the combination of alcohol consumption and an *ALDH2* polymorphism—found in rs671 A allele carriers—could be a risk factor for gastric cancer [149]. Alcohol consumption enhances acid secretion from the stomach, which leads to gastric mucosal damage [149] and the generation of ROS, subsequently promoting the carcinogenesis of gastric cancer [150]. Alcohol is endogenously broken down into acetaldehyde, which can produce DNA strand breakage and abnormal binding to proteins, potentially leading to cancer development [151]. A recent study has demonstrated that chronic alcohol consumption enhances intestinal tumorigenesis and tumor invasion and metastasis in genetically susceptible mice, as well as increases in polyp-associated mast cells and mast cell-mediated tumor migration [152], suggesting that mast cell-mediated inflammation could promote carcinogenesis [152].

DM increases the risk of colorectal cancer by up to three times greater than that of the general population, which makes colorectal cancer one of the most common cancers in patients with DM. It was reported that insulin and IGF-1 in DM promoted the phosphorylation of extracellular-signal-regulated kinase 1/2 (ERK1/2) and c-Jun N-terminal kinase (JNK). ERK1/2, P38, and JNK are the three major MAPK families found to be activated in colorectal cancer. These kinases increased B-cell lymphoma 2 (Bcl-2) and increased Bcl-2-associated X protein (Bax) expression, inducing anti-apoptosis and the proliferation of colon tumors [151].

There was a positive association between alcohol consumption and CRC risk in humans [153,154,155]. However, pre-diagnostic wine consumption is associated with more favorable survival after CRC [156]. It was also known that moderate wine consumption has been linked to lower levels of inflammatory markers [157]. Red wine contains resveratrol and polyphenols. Studies using mice found that resveratrol and its nano-formulation triggered apoptosis in cancer cell lines [158] and inhibited intestinal genes involved in cell proliferation or cell cycle progression [159,160]. Interestingly, CRC risk factors in patients vary by sex. Smoking and heavy alcohol consumption were significant risk factors of CRC in males compared to females [161]. However, female patients with a BMI ≥ 25 kg/m and abdominal obesity were at a higher risk of developing CRC than males [161].

## 8. Diabetes, Alcohol, and Bladder Cancer

Most studies have suggested a positive association between DM and a greater risk of bladder cancer morbidity and mortality, particularly in men [162,163,164]. A recent cumulative meta-analysis showed that DM was positively associated with bladder cancer mortality in both men and women [162]. Some studies have provided further evidence of a potential risk of bladder cancer associated with insulin. Insulin enhances bladder cancer cell growth by activating epidermal growth factor and PI3K pathways [165,166]. Chronic exposure to hyperinsulinemia or hyperglycemia induces tumor cell proliferation and metastasis, which increases insulin-like growth factor (IGF)-1 in diabetic patients, stimulates cellular proliferation, and inhibits apoptosis [167]. In DM patients with bladder cancer, the differential regulation of cadherin expression and the degradation of glycosaminoglycans were observed. Furthermore, reduced expression of E-cadherin was associated with poor outcomes in bladder cancer patients, which demonstrated an increase in metastasis.

The association between elevated plasma-free fatty acid (FFA) concentrations and insulin resistance has been demonstrated. Although the relationship between FFAs and insulin resistance is complex, a study demonstrated negative correlations between plasma FFA levels and the expression of peroxisome proliferator-activated receptor-gamma cofactor-1 (PGC-1) and nuclear-encoded mitochondrial genes. It was concluded that an increase in FFAs decreases the expression of PGC-1 and nuclear-encoded mitochondrial genes and also enhances the expression of extracellular matrix genes in a manner similar to those of inflammatory diseases [168].

A meta-analysis showed that alcohol consumption was not associated with bladder cancer [169]. However, another study conducted in the Netherlands found an elevated risk of bladder cancer in heavy drinkers [170,171]. This disparity could be attributed to gene–environment interactions, in which *ALDH2* (rs671, Glu504Lys) and *ADH1B* (rs1229984, His47Arg) polymorphisms showed the highest risk of bladder cancer, whereas among never-drinkers, no significant elevation of risk was observed with *ALDH2* Glu/Lys compared with Glu/Glu [172]. Furthermore, the concentration of acetaldehyde in urine 30–300 min after alcohol intake was 2–6 times higher among those with *ALDH2* Glu/Lys or Lys/Lys genotype than those with Glu/Glu [173], suggesting that heavily alcoholic individuals with *ALDH2* Glu/Lys are subject to prolonged exposure to acetaldehyde in urine. Acetaldehyde, by binding to DNA and cellular proteins, forms adducts, which can activate proto-oncogenes, inactivate tumor suppressor genes in replicating cells, and inhibit numerous important enzymes of DNA synthesis pathways [174].

## 9. Conclusions

Diabetes, obesity, alcoholism, smoking, chemical exposure, and dietary patterns are closely associated with cancer risk. Although DM and cancer share many common risk factors, several population-based retrospective cohort studies have demonstrated that DM may potentiate gastroenterological carcinogenesis. Thus, avoiding these risks will attenuate the morbidity and mortality of cancers. Furthermore, the discovery of new cancer-related genes is very important for cancer screening strategies and prevention. DM, alcohol misuse, and cancer attract public concern because they are not only medical and health issues but also represent social and financial burdens globally. Therefore, health authorities, social workers, and government agencies should manage DM, alcohol consumption, and cancer in such a way so that these diseases can be prevented and treated simultaneously. Eventually, a better comprehension of pathogenesis and novel therapies should be extensively studied to lower or eliminate DM, alcoholism, and cancer and save human lives.

## References

[1] McGinn O., Gupta V.K., Dauer P., Arora N., Sharma N., Nomura A., Dudeja V., Saluja A., Banerjee S. (2017). Inhibition of hypoxic response decreases stemness and reduces tumorigenic signaling due to impaired assembly of HIF1 transcription complex in pancreatic cancer. Sci. Rep..

[2] Kushwah V., Agrawal A.K., Dora C.P., Mallinson D., Lamprou D.A., Gupta R.C., Jain S. (2017). Novel Gemcitabine Conjugated Albumin Nanoparticles: A Potential Strategy to Enhance Drug Efficacy in Pancreatic Cancer Treatment. Pharm. Res..

[3] Seleznik G.M., Reding T., Peter L., Gupta A., Steiner S.G., Sonda S., Verbeke C.S., Dejardin E., Khatkov I., Segerer S. (2017). Development of autoimmune pancreatitis is independent of CDKN1A/p21-mediated pancreatic inflammation. Gut.

[4] Brooks P.J., Zakhari S. (2014). Acetaldehyde and the genome: Beyond nuclear DNA adducts and carcinogenesis. Environ. Mol. Mutagen..

[5] Srinivasan S., Dubey K.K., Singhal R.S. (2019). Influence of food commodities on hangover based on alcohol dehydrogenase and aldehyde dehydrogenase activities. Curr. Res. Food Sci..

[6] Schicchi A., Besson H., Rasamison R., Berleur M.-P., Mégarbane B. (2019). Fomepizole to treat disulfiram-ethanol reaction: A case series. Clin. Toxicol..

[7] Yoshimura Y., Nudelman A.S., Levery S.B., Wandall H.H., Bennett E.P., Hindsgaul O., Clausen H., Nishimura S. (2012). Elucidation of the sugar recognition ability of the lectin domain of UDP-GalNAc:polypeptide N-acetylgalactosaminyltransferase 3 by using unnatural glycopeptide substrates. Glycobiology.

[8] Hao M., Wang W., Zhao Y., Zhang R., Wang H. (2011). Pharmacokinetics and tissue distribution of 25-hydroxyprotopanaxadiol, an anti-cancer compound isolated from Panax ginseng, in athymic mice bearing xenografts of human pancreatic tumors. Eur. J. Drug Metab. Pharmacokinet..

[9] Sheridan H., Nestor C., O’Driscoll L., Hook I. (2011). Isolation, structure elucidation, and cytotoxic evaluation of furanonaphthoquinones from in vitro plantlets and cultures of Streptocarpus dunnii. J. Nat. Prod..

[10] Abe R. (2008). AGE-RAGE System and Carcinogenesis. Curr. Pharm. Des..

[11] Colditz G.A., Peterson L.L. (2018). Obesity and Cancer: Evidence, Impact, and Future Directions. Clin. Chem..

[12] Matsumoto K., Hasegawa T., Ohara K., Takei C., Kamei T., Koyanagi J., Takahashi T., Akimoto M. (2019). A metabolic pathway for the prodrug nabumetone to the pharmacologically active metabolite, 6-methoxy-2-naphthylacetic acid (6-MNA) by non-cytochrome P450 enzymes. Xenobiotica.

[13] Ye Y., Chen F., Wu H., Lan S.N., Jiang L.R., Dai K.K., Yan Y.Y., Yang L., Liao L.C. (2019). Relationship between Blood Acetaldehyde Concentration and Psychomotor Function of Individuals with Different ALDH2 Genotypes after Alcohol Consumption. Fa Yi Xue Za Zhi.

[14] Matsumura Y., Stiles K.M., Reid J., Frenk E.Z., Cronin S., Pagovich O.E., Crystal R.G. (2019). Gene Therapy Correction of Aldehyde Dehydrogenase 2 Deficiency. Mol. Ther. Methods Clin. Dev..

[15] Serio R.N., Lu C., Gross S.S., Gudas L.J. (2019). Different Effects of Knockouts in ALDH 2 and ACSS 2 on Embryonic Stem Cell Differentiation. Alcohol. Clin. Exp. Res..

[16] Lee Y.J., Yoo M.G., Kim H.K., Jang H.B., Park K.J., Lee H.J., Kim S.G., Park S.I. (2019). The association between alcohol metabolism and genetic variants of ADH1A, SRPRB, and PGM1 in Korea. Alcohol.

[17] Fu X., Yang H., Pangestu F., Nikolau B.J. (2020). Failure to Maintain Acetate Homeostasis by Acetate-Activating Enzymes Impacts Plant Development. Plant Physiol..

[18] Ouyang D.W., Ni X., Xu H.Y., Chen J., Yang P.M., Kong D.Y. (2010). Pterosins from Pteris multifida. Planta Med..

[19] Li C., Yang Z., Zhai C., Qiu W., Li D., Yi Z., Wang L., Tang J., Qian M., Luo J. (2010). Maslinic acid potentiates the anti-tumor activity of tumor necrosis factor alpha by inhibiting NF-kappaB signaling pathway. Mol. Cancer.

[20] Viswesh V., Gates K., Sun D. (2010). Characterization of DNA damage induced by a natural product antitumor antibiotic leinamycin in human cancer cells. Chem. Res. Toxicol..

[21] Rawla P., Sunkara T., Gaduputi V. (2019). Epidemiology of Pancreatic Cancer: Global Trends, Etiology and Risk Factors. World J. Oncol..

[22] Capasso M., Franceschi M., Rodriguez-Castro K.I., Crafa P., Cambiè G., Miraglia C., Barchi A., Nouvenne A., Leandro G., Meschi T. (2018). Epidemiology and risk factors of pancreatic cancer. Acta BioMed Atenei Parm..

[23] Lugo A., Peveri G., Bosetti C., Bagnardi V., Crippa A., Orsini N., Rota M., Gallus S. (2018). Strong excess risk of pancreatic cancer for low frequency and duration of cigarette smoking: A comprehensive review and meta-analysis. Eur. J. Cancer.

[24] Bellastella G., Scappaticcio L., Esposito K., Giugliano D., Maiorino M.I. (2018). Metabolic syndrome and cancer: “The common soil hypothesis”. Diabetes Res. Clin. Pr..

[25] Baecker A., Liu X., La Vecchia C., Zhang Z.-F. (2018). Worldwide incidence of hepatocellular carcinoma cases attributable to major risk factors. Eur. J. Cancer Prev..

[26] Clements O., Eliahoo J., Kim J.U., Taylor-Robinson S.D., Khan S.A. (2020). Risk factors for intrahepatic and extrahepatic cholangiocarcinoma: A systematic review and meta-analysis. J. Hepatol..

[27] Sagnelli E., Macera M., Russo A., Coppola N., Sagnelli C. (2020). Epidemiological and etiological variations in hepatocellular carcinoma. Infection.

[28] López-Suárez A. (2019). Burden of cancer attributable to obesity, type 2 diabetes and associated risk factors. Metabolism.

[29] Gupta V.K., Banerjee S. (2017). Isolation of Lipid Raft Proteins from CD133+ Cancer Stem Cells. Methods Mol. Biol..

[30] Papalazarou C., Klop G.J., Milder M.T., Marijnissen J.P., Gupta V., Heijmen B.J., Nuyttens J.J., Hoogeman M.S. (2017). CyberKnife with integrated CT-on-rails: System description and first clinical application for pancreas SBRT. Med. Phys..

[31] Siegel R.L., Miller K.D., Jemal A. (2020). Cancer statistics, 2020. CA Cancer J. Clin..

[32] Leung T.-M., Nieto N. (2013). CYP2E1 and oxidant stress in alcoholic and non-alcoholic fatty liver disease. J. Hepatol..

[33] Elamin E.E., Masclee A., Dekker J., Jonkers D. (2013). Ethanol metabolism and its effects on the intestinal epithelial barrier. Nutr. Rev..

[34] Poschl G. (2004). Alcohol and cancer. Lancet Alcohol..

[35] Väkeväinen S., Mentula S., Nuutinen H., Salmela K.S., Jousimies-Somer H., Färkkilä M., Salaspuro M. (2002). Ethanol-derived microbial production of carcinogenic acetaldehyde in achlorhydric atrophic gastritis. Scand. J. Gastroenterol..

[36] Gong J., Xie J., Bedolla R., Rivas P., Chakravarthy D., Freeman J.W., Reddick R., Kopetz S., Peterson A., Wang H. (2014). Combined targeting of STAT3/NF-κB/COX-2/EP4 for effective management of pancreatic cancer. Clin. Cancer Res..

[37] Seo W., Gao Y., He Y., Sun J., Xu H., Feng D., Park S.H., Cho Y.-E., Guillot A., Ren T. (2019). ALDH2 deficiency promotes alcohol-associated liver cancer by activating oncogenic pathways via oxidized DNA-enriched extracellular vesicles. J. Hepatol..

[38] Yu W., Ma Y., Shankar S., Srivastava R.K. (2018). Chronic ethanol exposure of human pancreatic normal ductal epithelial cells induces cancer stem cell phenotype through SATB2. J. Cell. Mol. Med..

[39] Serio R.N., Gudas L.J. (2020). Modification of stem cell states by alcohol and acetaldehyde. Chem. Interact..

[40] Larocque K., Ovadje P., Djurdjevic S., Mehdi M., Green J., Pandey S. (2014). Novel Analogue of Colchicine Induces Selective Pro-Death Autophagy and Necrosis in Human Cancer Cells. PLoS ONE.

[41] Leboeuf C., Mailly L., Wu T., Bour G., Durand S., Brignon N., Ferrand C., Borg C., Tiberghien P., Thimme R. (2014). In Vivo Proof of Concept of Adoptive Immunotherapy for Hepatocellular Carcinoma Using Allogeneic Suicide Gene-modified Killer Cells. Mol. Ther..

[42] Breeden J.H. (1984). Alcohol, Alcoholism, and Cancer. Med. Clin. North Am..

[43] Herreros-Villanueva M., Hijona E., Bañales J.M., Cosme A., Bujanda L. (2013). Alcohol consumption on pancreatic diseases. World J. Gastroenterol..

[44] Kocher H.M., Alrawashdeh W. (2010). Pancreatic cancer. BMJ Clin. Evid..

[45] Ringborg U. (1998). Alcohol and Risk of Cancer. Alcohol Clin. Exp. Res..

[46] Stermer E. (2002). Alcohol consumption and the gastrointestinal tract. Isr. Med. Assoc. J. IMAJ.

[47] Tan H.K., Yates E., Lilly K., Dhanda A.D. (2020). Oxidative stress in alcohol-related liver disease. World J. Hepatol..

[48] Wang W., Wang C., Xu H., Gao Y. (2020). Aldehyde Dehydrogenase, Liver Disease and Cancer. Int. J. Biol. Sci..

[49] Pöschl G., Stickel F., Wang X.D., Seitz H.K. (2004). Alcohol and cancer: Genetic and nutritional aspects. Proc. Nutr. Soc..

[50] De Hert M., Peuskens J., Sabbe T., Mitchell A.J., Stubbs B., Neven P., Wildiers H., Detraux J. (2015). Relationship between prolactin, breast cancer risk, and antipsychotics in patients with schizophrenia: A critical review. Acta Psychiatr. Scand..

[51] Widmer R.J., Flammer A.J., Lerman L.O., Lerman A. (2015). The Mediterranean Diet, its Components, and Cardiovascular Disease. Am. J. Med..

[52] De Lorgeril M., Salen P. (2014). Do statins increase and Mediterranean diet decrease the risk of breast cancer?. BMC Med..

[53] Maskarinec G., Jacobs S., Park S.-Y., Haiman C.A., Setiawan V., Wilkens L.R., Le Marchand L. (2017). Type II Diabetes, Obesity, and Breast Cancer Risk: The Multiethnic Cohort. Cancer Epidemiol. Biomark. Prev..

[54] Michels K.B., Solomon C.G., Hu F.B., Rosner B.A., Hankinson S.E., Colditz G.A., Manson J.E. (2003). Type 2 Diabetes and Subsequent Incidence of Breast Cancer in the Nurses’ Health Study. Diabetes Care.

[55] Woźniak M.K., Wiergowski M., Namieśnik J., Biziuk M. (2019). Biomarkers of Alcohol Consumption in Body Fluids—Possibilities and Limitations of Application in Toxicological Analysis. Curr. Med. Chem..

[56] Chen J., Qin C., Zhou Y., Chen Y., Mao M., Yang J. (2021). Metformin may induce ferroptosis by inhibiting autophagy via lncRNA H19 in breast cancer. FEBS Open Bio..

[57] Shi B., Hu X., He H., Fang W. (2021). Metformin suppresses breast cancer growth via inhibition of cyclooxygenase-2. Oncol. Lett..

[58] Yenmiş G., Beşli N., Saraç E.Y., Emre F.S.H., Şenol K., Sultuybek G.K. (2021). Metformin promotes apoptosis in primary breast cancer cells by downregulation of cyclin D1 and upregulation of P53 through an AMPK-alpha independent mechanism. Turk. J. Med. Sci..

[59] Dam M.K., Hvidtfeldt U.A., Tjonneland A., Overvad K., Grønbæk M.K., Tolstrup J. (2016). Five year change in alcohol intake and risk of breast cancer and coronary heart disease among postmenopausal women: Prospective cohort study. BMJ.

[60] Leung T., Rajendran R., Singh S., Garva R., Krstic-Demonacos M., Demonacos C. (2013). Cytochrome P450 2E1 (CYP2E1) regulates the response to oxidative stress and migration of breast cancer cells. Breast Cancer Res..

[61] Larsen S.B., Kroman N., Ibfelt E.H., Christensen J., Tjønneland A., Dalton S.O. (2015). Influence of metabolic indicators, smoking, alcohol and socioeconomic position on mortality after breast cancer. Acta Oncol..

[62] Steele V.E., Lubet R.A. (2010). The Use of Animal Models for Cancer Chemoprevention Drug Development. Semin. Oncol..

[63] Heretsch P., Tzagkaroulaki L., Giannis A. (2010). Modulators of the hedgehog signaling pathway. Bioorg. Med. Chem..

[64] Kumar D., Singh G., Sharma P., Qayum A., Mahajan G., Mintoo M., Singh S.K., Mondhe D.M., Bedi P., Jain S.K. (2018). 4-aryl/heteroaryl-4H-fused Pyrans as Anti-proliferative Agents: Design, Synthesis and Biological Evaluation. Anti-Cancer Agents Med. Chem..

[65] Poudel B.K., Gupta B., Ramasamy T., Thapa R.K., Pathak S., Oh K.T., Jeong J.-H., Choi H.-G., Yong C.S., Kim J.O. (2017). PEGylated thermosensitive lipid-coated hollow gold nanoshells for effective combinational chemo-photothermal therapy of pancreatic cancer. Colloids Surfaces B Biointerfaces.

[66] Goodenberger M.H., Wagner-Bartak N.A., Gupta S., Liu X., Yap R.Q., Sun J., Tamm E.P., Jensen C.T. (2018). Computed Tomography Image Quality Evaluation of a New Iterative Reconstruction Algorithm in the Abdomen (Adaptive Statistical Iterative Reconstruction–V) a Comparison With Model-Based Iterative Reconstruction, Adaptive Statistical Iterative Reconstruction, and Filtered Back Projection Reconstructions. J. Comput. Assist. Tomogr..

[67] Gupta R., Amanam I., Chung V. (2017). Current and future therapies for advanced pancreatic cancer. J. Surg. Oncol..

[68] Vashi P.G., Virginkar N., Popiel B., Edwin P., Gupta D. (2017). Incidence of and factors associated with catheter-related bloodstream infection in patients with advanced solid tumors on home parenteral nutrition managed using a standardized catheter care protocol. BMC Infect. Dis..

[69] Gupta N., Rath S.K., Singh J., Qayum A., Singh S., Sangwan P.L. (2017). Synthesis of novel benzylidene analogues of betulinic acid as potent cytotoxic agents. Eur. J. Med. Chem..

[70] Dauer P., Gupta V.K., McGinn O., Nomura A., Sharma N., Arora N., Giri B., Dudeja V., Saluja A.K., Banerjee S. (2017). Inhibition of Sp1 prevents ER homeostasis and causes cell death by lysosomal membrane permeabilization in pancreatic cancer. Sci. Rep..

[71] Dangroo N.A., Singh J., Rath S.K., Gupta N., Qayum A., Singh S., Sangwan P.L. (2017). A convergent synthesis of novel alkyne–azide cycloaddition congeners of betulinic acid as potent cytotoxic agent. Steroids.

[72] Fomenko E.V., Chi Y. (2016). Mangiferin modulation of metabolism and metabolic syndrome. BioFactors.

[73] Stratton-Powell A.A., Pasko K.M., Brockett C.L., Tipper J.L. (2016). The Biologic Response to Polyetheretherketone (PEEK) Wear Particles in Total Joint Replacement: A Systematic Review. Clin. Orthop. Relat. Res..

[74] Wang S., Pacher P., De Lisle R.C., Huang H., Ding W.-X. (2016). A Mechanistic Review of Cell Death in Alcohol-Induced Liver Injury. Alcohol. Clin. Exp. Res..

[75] Engel M.F., Muijsken M.A., Mooi-Kokenberg E., Kuijper E., Van Westerloo D.J. (2016). Vibrio cholerae non-O1 bacteraemia: Description of three cases in the Netherlands and a literature review. Eurosurveillance.

[76] Takada S., Fujiwara S., Inoue T., Kataoka Y., Hadano Y., Matsumoto K., Morino K., Shimizu T. (2016). Meningococcemia in Adults: A Review of the Literature. Intern. Med..

[77] Shepherd S., Newman R., Brett S.J., Griffith D.M. (2016). Pharmacological Therapy for the Prevention and Treatment of Weakness After Critical Illness. Crit. Care Med..

[78] Leonard E.A., Buckley T., Curtis K. (2016). Impact of Alcohol on Outcomes in Hospitalized Major Trauma Patients. J. Trauma Nurs..

[79] Guerrero M., Urbano M., Roberts E. (2016). Sphingosine 1-phosphate receptor 1 agonists: A patent review (2013-2015). Expert Opin. Ther. Patents.

[80] Hemalatha K., Madhumitha G. (2016). Inhibition of poly(adenosine diphosphate-ribose) polymerase using quinazolinone nucleus. Appl. Microbiol. Biotechnol..

[81] Younus H., Anwar S. (2016). Prevention of Non-Enzymatic Glycosylation (Glycation): Implication in the Treatment of Diabetic Complication. Int. J. Health Sci..

[82] Liu C.-S., Zheng Y.-R., Zhang Y.-F., Long X.-Y. (2016). Research progress on berberine with a special focus on its oral bioavailability. Fitoterapia.

[83] Wang Y.-S., Wen Z.-Q., Li B.-T., Zhang H.-B., Yang J.-H. (2016). Ethnobotany, phytochemistry, and pharmacology of the genus Litsea: An update. J. Ethnopharmacol..

[84] Balaji M., Ganjayi M.S., Kumar G.E.H., Parim B.N., Mopuri R., Dasari S. (2016). A review on possible therapeutic targets to contain obesity: The role of phytochemicals. Obes. Res. Clin. Pr..

[85] Ramasamy R., Shekhtman A., Schmidt A.M. (2016). The multiple faces of RAGE—opportunities for therapeutic intervention in aging and chronic disease. Expert Opin. Ther. Targets.

[86] Shukla R., Sharma D.C., Baig M.H., Bano S., Roy S., Provazník I., Kamal M.A. (2016). Antioxidant, Antimicrobial Activity and Medicinal Properties of Grewia asiatica L.. Med. Chem..

[87] Michaud D.S. (2016). Epidemiology of Pancreatic Cancer. Pathol. Epidemiol. Cancer.

[88] Siegel R.L., Miller K.D., Jemal A. (2017). Cancer statistics, 2017. CA: A Cancer J. Clin..

[89] Raghow R. (2013). Betatrophin: A liver-derived hormone for the pancreatic β-cell proliferation. World J. Diabetes.

[90] Yi K.H., Hwang J.S., Kim E.Y., Lee S.H., Kim D.H., Lim J.S. (2014). Prevalence of insulin resistance and cardiometabolic risk in Korean children and adolescents: A population-based study. Diabetes Res. Clin. Pr..

[91] Manivannan E., Amawi H., Hussein N., Karthikeyan C., Fetcenko A., Moorthy N.H.N., Trivedi P., Tiwari A.K. (2017). Design and discovery of silybin analogues as antiproliferative compounds using a ring disjunctive—Based, natural product lead optimization approach. Eur. J. Med. Chem..

[92] Lee S.Y., Kim W., Lee Y.G., Kang H.J., Lee S.-H., Park S.Y., Min J.-K., Lee S.-R., Chung S.J. (2017). Identification of sennoside A as a novel inhibitor of the slingshot (SSH) family proteins related to cancer metastasis. Pharmacol. Res..

[93] Bi Y., Shen W., Min M., Liu Y. (2017). MicroRNA-7 functions as a tumor-suppressor gene by regulating ILF2 in pancreatic carcinoma. Int. J. Mol. Med..

[94] Harshbarger W., Gondi S., Ficarro S.B., Hunter J., Udayakumar D., Gurbani D., Singer W.D., Liu Y., Li L., Marto J.A. (2017). Structural and Biochemical Analyses Reveal the Mechanism of Glutathione S-Transferase Pi 1 Inhibition by the Anti-cancer Compound Piperlongumine. J. Biol. Chem..

[95] Boukes G.J., Van De Venter M. (2016). The apoptotic and autophagic properties of two natural occurring prodrugs, hyperoside and hypoxoside, against pancreatic cancer cell lines. Biomed. Pharmacother..

[96] Arora D., Sharma N., Singamaneni V., Sharma V., Kushwaha M., Abrol V., Guru S., Sharma S., Gupta A.P., Bhushan S. (2016). Isolation and characterization of bioactive metabolites from Xylaria psidii, an endophytic fungus of the medicinal plant Aegle marmelos and their role in mitochondrial dependent apoptosis against pancreatic cancer cells. Phytomedicine.

[97] Zhang T., Li S., Li J., Yin F., Hua Y., Wang Z., Lin B., Wang H., Zou D., Zhou Z. (2016). RETRACTED ARTICLE: Natural product pectolinarigenin inhibits osteosarcoma growth and metastasis via SHP-1-mediated STAT3 signaling inhibition. Cell Death Dis..

[98] Duan H., Lee J.W., Moon S.W., Arora D., Li Y., Lim H.-Y., Wang W. (2016). Discovery, Synthesis, and Evaluation of 2,4-Diaminoquinazolines as a Novel Class of Pancreatic β-Cell-Protective Agents against Endoplasmic Reticulum (ER) Stress. J. Med. Chem..

[99] Guzmán E.A., Xu Q., Pitts T.P., Mitsuhashi K.O., Baker C., Linley P.A., Oestreicher J., TenDyke K., Winder P.L., Suh E.M. (2016). Leiodermatolide, a novel marine natural product, has potent cytotoxic and antimitotic activity against cancer cells, appears to affect microtubule dynamics, and exhibits antitumor activity. Int. J. Cancer.

[100] Kasukabe T., Honma Y., Okabe-Kado J., Higuchi Y., Kato N., Kumakura S. (2016). Combined treatment with cotylenin A and phenethyl isothiocyanate induces strong antitumor activity mainly through the induction of ferroptotic cell death in human pancreatic cancer cells. Oncol. Rep..

[101] Kumar V., Guru S.K., Jain S.K., Joshi P., Gandhi S.G., Bharate S.B., Bhushan S., Bharate S.S., Vishwakarma R.A. (2016). A chromatography-free isolation of rohitukine from leaves of Dysoxylum binectariferum: Evaluation for in vitro cytotoxicity, Cdk inhibition and physicochemical properties. Bioorg. Med. Chem. Lett..

[102] Chakraborty S., Rasool R.U., Kumar S., Nayak D., Rah B., Katoch A., Amin H., Ali A., Goswami A. (2016). Cristacarpin promotes ER stress-mediated ROS generation leading to premature senescence by activation of p21waf-1. AGE.

[103] Huang K.-C., Chen Z., Jiang Y., Akare S., Kolber-Simonds D., Condon K., Agoulnik S., TenDyke K., Shen Y., Wu K.-M. (2016). Apratoxin A Shows Novel Pancreas-Targeting Activity through the Binding of Sec 61. Mol. Cancer Ther..

[104] Xie Y., Song X., Sun X., Huang J., Zhong M., Lotze M.T., Zeh H.J., Kang R., Tang D. (2016). Identification of baicalein as a ferroptosis inhibitor by natural product library screening. Biochem. Biophys. Res. Commun..

[105] Koul M., Meena S., Kumar A., Sharma P.R., Singamaneni V., Riyaz-Ul-Hassan S., Hamid A., Chaubey A., Prabhakar A., Gupta P. (2016). Secondary Metabolites from Endophytic Fungus Penicillium pinophilum Induce ROS-Mediated Apoptosis through Mitochondrial Pathway in Pancreatic Cancer Cells. Planta Med..

[106] Farley C., Dibwe D.F., Ueda J.-Y., Hall E.A., Awale S., Magolan J. (2016). Evaluation of synthetic coumarins for antiausterity cytotoxicity against pancreatic cancers. Bioorg. Med. Chem. Lett..

[107] Kurkivuori J., Salaspuro V., Kaihovaara P., Kari K., Rautemaa R., Grönroos L., Meurman J., Salaspuro M. (2007). Acetaldehyde production from ethanol by oral streptococci. Oral Oncol..

[108] Bar M., Burke M., Isakov A., Almog C. (1990). Insulinoma after streptozotocin therapy for metastatic gastrinoma: Natural history or iatrogenic complication?. J. Clin. Gastroenterol..

[109] Spiess J., Rivier J., Thorner M., Vale W. (1982). Sequence analysis of a growth hormone releasing factor from a human pancreatic islet tumor. Biochemistry.

[110] Gounder V.K., Arumugam S., Giridharan V.V., Sreedhar R., Bose R.J., Vanama J., Palaniyandi S.S., Konishi T., Watanabe K., Thandavarayan R.A. (2017). Tiny molecule, big power: Multi-target approach for curcumin in diabetic cardiomyopathy. Nutrition.

[111] Behl T., Kotwani A. (2017). Proposed mechanisms of Terminalia catappa in hyperglycaemia and associated diabetic complications. J. Pharm. Pharmacol..

[112] Ezzikouri S., Jadid F.Z., Hamdi S., Wakrim L., Tsukiyama-Kohara K., Benjelloun S. (2016). Supplementing Conventional Treatment with Pycnogenol® May Improve Hepatitis C Virus–Associated Type 2 Diabetes: A Mini Review. J. Clin. Transl. Hepatol..

[113] Parkin D.M. (2006). The global health burden of infection-associated cancers in the year 2002. Int. J. Cancer.

[114] Vatamaniuk M.Z., Gupta R.K., Lantz K.A., Doliba N.M., Matschinsky F.M., Kaestner K.H. (2006). Foxa1-Deficient Mice Exhibit Impaired Insulin Secretion due to Uncoupled Oxidative Phosphorylation. Diabetes.

[115] Chuang S.-C., Lee Y.-C.A., Wu G.-J., Straif K., Hashibe M. (2015). Alcohol consumption and liver cancer risk: A meta-analysis. Cancer Causes Control..

[116] Taniai M. (2020). Alcohol and hepatocarcinogenesis. Clin. Mol. Hepatol..

[117] Thylur R.P., Roy S.K., Shrivastava A., LaVeist T.A., Shankar S., Srivastava R.K. (2020). Assessment of risk factors, and racial and ethnic differences in hepatocellular carcinoma. JGH Open.

[118] Makol A., Kanthaje S., Dhiman R.K., Kalra N., Chawla Y.K., Chakraborti A. (2017). Association of liver cirrhosis severity with type 2 diabetes mellitus in hepatocellular carcinoma. Exp. Biol. Med..

[119] Lim H.-W., Bernstein D.E. (2018). Risk Factors for the Development of Nonalcoholic Fatty Liver Disease/Nonalcoholic Steatohepatitis, Including Genetics. Clin. Liver Dis..

[120] Rota M., Bellocco R., Scotti L., Tramacere I., Jenab M., Corrao G., La Vecchia C., Boffetta P., Bagnardi V. (2010). Random-effects meta-regression models for studying nonlinear dose-response relationship, with an application to alcohol and esophageal squamous cell carcinoma. Stat. Med..

[121] Turati F., Garavello W., Tramacere I., Bagnardi V., Rota M., Scotti L., Islami F., Corrao G., Boffetta P., La Vecchia C. (2010). A meta-analysis of alcohol drinking and oral and pharyngeal cancers. Part 2: Results by subsites. Oral Oncol..

[122] Tramacere I., Negri E., Bagnardi V., Garavello W., Rota M., Scotti L., Islami F., Corrao G., Boffetta P., La Vecchia C. (2010). A meta-analysis of alcohol drinking and oral and pharyngeal cancers. Part 1: Overall results and dose-risk relation. Oral Oncol..

[123] Turati F., Gallus S., Tavani A., Tramacere I., Polesel J., Talamini R., Montella M., Scotti L., Franceshi S., La Vecchia C. (2010). Alcohol and endometrial cancer risk: A case–control study and a meta-analysis. Cancer Causes Control..

[124] Tramacere I., Scotti L., Jenab M., Bagnardi V., Bellocco R., Rota M., Corrao G., Bravi F., Boffetta P., La Vecchia C. (2009). Alcohol drinking and pancreatic cancer risk: A meta-analysis of the dose-risk relation. Int. J. Cancer.

[125] Genkinger J.M., Spiegelman D., Anderson K.E., Bergkvist L., Bernstein L., Brandt P.V.D., English D., Freudenheim J.L., Fuchs C.S., Giles G. (2009). Alcohol Intake and Pancreatic Cancer Risk: A Pooled Analysis of Fourteen Cohort Studies. Cancer Epidemiol. Biomark. Prev..

[126] Berchtold A., Akre C., Jeannin A., Michaud P.-A., Suris J.-C. (2011). First consumption ever of multiple substances: Applying an expert-based taxonomy to a Swiss national sample of adolescents. Addict. Behav..

[127] Meier M., Berchtold A., Akré C., Michaud P.-A., Surís J.-C. (2010). Who eats healthily? A population-based study among young Swiss residents. Public Health Nutr..

[128] Michaud D.S., Vrieling A., Jiao L., Mendelsohn J.B., Steplowski E., Lynch S.M., Wactawski-Wende J., Arslan A., Bueno-De-Mesquita H.B., Fuchs C.S. (2010). Alcohol intake and pancreatic cancer: A pooled analysis from the pancreatic cancer cohort consortium (PanScan). Cancer Causes Control..

[129] Gmel G., Gaume J., Willi C., Michaud P.-A., Cornuz J., Daeppen J.-B. (2010). Challenging the “Inoffensiveness” of Regular Cannabis Use by Its Associations with Other Current Risky Substance Use—A Census of 20-Year-Old Swiss Men. Int. J. Environ. Res. Public Health.

[130] Brock B., Gregersen S., Kristensen K., Thomsen J.L., Buschard K., Kofod H., Hermansen K. (1999). The insulinotropic effect of endothelin-1 is mediated by glucagon release from the islet alpha cells. Diabetologia.

[131] Thomsen C., Rasmussen O., Lousen T., Holst J.J., Fenselau S., Schrezenmeir J., Hermansen K. (1999). Differential effects of saturated and monounsaturated fatty acids on postprandial lipemia and incretin responses in healthy subjects. Am. J. Clin. Nutr..

[132] Kudahl A.K., Søren G., Majgaard J.H., Laust T.J., Hermansen K. (1999). Differential effects of cis and trans fatty acids on insulin release from isolated mouse islets. Metabolism.

[133] Seitz H.K., Stickel F. (2006). Risk factors and mechanisms of hepatocarcinogenesis with special emphasis on alcohol and oxidative stress. Biol. Chem..

[134] Siegel A.B., Goyal A., Salomao M., Wang S., Lee V., Hsu C., Rodriguez R., Hershman D.L., Brown R.S., Neugut A.I. (2014). Serum Adiponectin Is Associated with Worsened Overall Survival in a Prospective Cohort of Hepatocellular Carcinoma Patients. Oncology.

[135] Correa P., Piazuelo M.B. (2011). Helicobacter pylori Infection and Gastric Adenocarcinoma. US Gastroenterol. Hepatol. Rev..

[136] Tian T., Zhang L.Q., Ma X.H., Zhou J.N., Shen J. (2012). Diabetes Mellitus and Incidence and Mortality of Gastric Cancer: A Meta-Analysis. Exp. Clin. Endocrinol. Diabetes.

[137] Yoon J.M., Son K.Y., Eom C.S., Durrance D., Park S.M. (2013). Pre-existing diabetes mellitus increases the risk of gastric cancer: A meta-analysis. World J. Gastroenterol..

[138] Ge Z., Ben Q., Qian J., Wang Y., Li Y. (2011). Diabetes mellitus and risk of gastric cancer. Eur. J. Gastroenterol. Hepatol..

[139] Jayaprakash V., Marimuthu S.P., Vijayaragavan P., Moysich K.B. (2011). Diabetes mellitus and gastric carcinoma: Is there an association?. J. Carcinog..

[140] Sekikawa A., Fukui H., Maruo T., Tsumura T., Okabe Y., Osaki Y. (2014). Diabetes mellitus increases the risk of early gastric cancer development. Eur. J. Cancer.

[141] Ikeda K., Kobayashi M., Someya T., Saitoh S., Hosaka T., Akuta N., Suzuki F., Suzuki Y., Arase Y., Kumada H. (2009). Occult hepatitis B virus infection increases hepatocellular carcinogenesis by eight times in patients with non-B, non-C liver cirrhosis: A cohort study. J. Viral Hepat..

[142] Kountouras J., Boura P., Lygidakis N.J. (2000). Omeprazole and regulation of cytokine profile in Helicobacter pylori-infected patients with duodenal ulcer disease. Hepatogastroenterology.

[143] Dandona P., Thusu K., Cook S., Snyder B., Makowski J., Armstrong D., Nicotera T. (1996). Oxidative damage to DNA in diabetes mellitus. Lancet.

[144] Vigneri R. (2009). Diabetes therapy and cancer risk. Nat. Rev. Endocrinol..

[145] Novosyadlyy R., Leroith D. (2010). Hyperinsulinemia and type 2 diabetes: Impact on cancer. Cell Cycle.

[146] Pollak M. (2008). Targeting insulin and insulin-like growth factor signalling in oncology. Curr. Opin. Pharmacol..

[147] Ma K., Baloch Z., He T.-T., Xia X. (2017). Alcohol Consumption and Gastric Cancer Risk: A Meta-Analysis. Med Sci. Monit..

[148] Yang C.S., Chen X., Tu S. (2016). Etiology and Prevention of Esophageal Cancer. Gastrointest. Tumors.

[149] Liu J., Yang H.-I., Lee M.-H., Jen C.-L., Hu H.-H., Lu S.-N., Wang L.-Y., You S.-L., Huang Y.-T., Chen C.-J. (2016). Alcohol Drinking Mediates the Association between Polymorphisms of ADH1B and ALDH2 and Hepatitis B–Related Hepatocellular Carcinoma. Cancer Epidemiol. Biomark. Prev..

[150] Fouad A.A., Al-Sultan A.I., Yacoubi M.T., Gomaa W. (2010). Ameliorative effects of telmisartan in diabetic rats with indomethacin-induced gastric ulceration. Eur. J. Pharmacol..

[151] Yacoub R., Habib H., Lahdo A., Al Ali R., Varjabedian L., Atalla G., Akl N.K., Aldakheel S., Alahdab S., Albitar S. (2010). Association between smoking and chronic kidney disease: A case control study. BMC Public Health.

[152] Wimberly A.L., Forsyth C.B., Khan M.W., Pemberton A., Khazaie K., Keshavarzian A. (2013). Ethanol-Induced Mast Cell-Mediated Inflammation Leads to Increased Susceptibility of Intestinal Tumorigenesis in the APCΔ468Min Mouse Model of Colon Cancer. Alcohol. Clin. Exp. Res..

[153] Huxley R.R., Ansary-Moghaddam A., Clifton P., Czernichow S., Parr C.L., Woodward M. (2009). The impact of dietary and lifestyle risk factors on risk of colorectal cancer: A quantitative overview of the epidemiological evidence. Int. J. Cancer.

[154] Kim D.-H., Ahn Y.-O. (2004). Molecular Epidemiology of Colon Cancer. Cancer Res. Treat..

[155] Le Marchand L., Wilkens L.R., Kolonel L.N., Hankin J.H., Lyu L.C. (1997). Associations of sedentary lifestyle, obesity, smoking, alcohol use, and diabetes with the risk of colorectal cancer. Cancer Res..

[156] McFarland M.S., Cripps R. (2010). Diabetes Mellitus and Increased Risk of Cancer: Focus on Metformin and the Insulin Analogs. Pharmacother. J. Hum. Pharmacol. Drug Ther..

[157] McFarland M.S., Knight T.N., Brown A., Thomas J. (2010). The Continuation of Oral Medications with the Initiation of Insulin Therapy in Type 2 Diabetes: A Review of the Evidence. South. Med. J..

[158] Hahn M., Sriharan K., McFarland M.S. (2010). Gemfibrozil-Induced Myositis in a Patient with Normal Renal Function. Ann. Pharmacother..

[159] Sudha T., El-Far A.H., Mousa D.S., Mousa S.A. (2020). Resveratrol and Its Nanoformulation Attenuate Growth and the Angiogenesis of Xenograft and Orthotopic Colon Cancer Models. Molecules.

[160] Sale S., Verschoyle R.D., Boocock D., Jones D., Wilsher N., Ruparelia K.C., Potter G.A., Farmer P.B., Steward W.P., Gescher A.J. (2004). Pharmacokinetics in mice and growth-inhibitory properties of the putative cancer chemopreventive agent resveratrol and the synthetic analogue trans 3,4,5,4′-tetramethoxystilbene. Br. J. Cancer.

[161] Lee J.M., Lee K.-M., Kim D.B., Ko S.-H., Park Y.G. (2019). Colorectal Cancer Risks According to Sex Differences in Patients With Type II Diabetes Mellitus. Clin. Transl. Gastroenterol..

[162] Zhu Z., Zhang X., Shen Z., Zhong S., Wang X., Lu Y., Xu C. (2013). Diabetes Mellitus and Risk of Bladder Cancer: A Meta-Analysis of Cohort Studies. PLoS ONE.

[163] Newton C.C., Gapstur S.M., Campbell P.T., Jacobs E.J. (2013). Type 2 diabetes mellitus, insulin-use and risk of bladder cancer in a large cohort study. Int. J. Cancer.

[164] Xu Y., Huo R., Chen X., Yu X. (2017). Diabetes mellitus and the risk of bladder cancer. Medicine.

[165] Ornskov D., Nexo E., Sorensen B.S. (2007). Insulin induces a transcriptional activation of epiregulin, HB-EGF and amphiregulin, by a PI3K-dependent mechanism: Identification of a specific insulin-responsive promoter element. Biochem. Biophys. Res. Commun..

[166] Ornskov D., Nexo E., Sorensen B.S. (2006). Insulin-induced proliferation of bladder cancer cells is mediated through activation of the epidermal growth factor system. FEBS J..

[167] Richardson D.W., Vinik A.I. (2005). Metabolic Implications of Obesity: Before and After Gastric Bypass. Gastroenterol. Clin. N. Am..

[168] Richardson D.K., Kashyap S., Bajaj M., Cusi K., Mandarino S.J., Finlayson J., DeFronzo R.A., Jenkinson C.P., Mandarino L.J. (2005). Lipid Infusion Decreases the Expression of Nuclear Encoded Mitochondrial Genes and Increases the Expression of Extracellular Matrix Genes in Human Skeletal Muscle. J. Biol. Chem..

[169] Vartolomei M.D., Iwata T., Roth B., Kimura S., Mathieu R., Ferro M., Shariat S., Seitz C. (2019). Impact of alcohol consumption on the risk of developing bladder cancer: A systematic review and meta-analysis. World J. Urol..

[170] Zaitsu M., Nakamura F., Toyokawa S., Tonooka A., Takeuchi T., Homma Y., Kobayashi Y. (2016). Risk of Alcohol Consumption in Bladder Cancer: Case-Control Study from a Nationwide Inpatient Database in Japan. Tohoku J. Exp. Med..

[171] Zeegers M.P.A., Volovics A., Dorant E., Goldbohm R.A., Brandt P.V.D. (2001). Alcohol Consumption and Bladder Cancer Risk: Results from the Netherlands Cohort Study. Am. J. Epidemiol..

[172] Masaoka H., Ito H., Soga N., Hosono S., Oze I., Watanabe M., Tanaka H., Yokomizo A., Hayashi N., Eto M. (2016). Aldehyde dehydrogenase 2 (ALDH2) and alcohol dehydrogenase 1B (ADH1B) polymorphisms exacerbate bladder cancer risk associated with alcohol drinking: Gene–environment interaction. Carcinogenesis.

[173] Otsuka M., Harada N., Itabashi T., Ohmori S. (1999). Blood and Urinary Levels of Ethanol, Acetaldehyde, and C4 Compounds Such as Diacetyl, Acetoin, and 2,3-Butanediol in Normal Male Students After Ethanol Ingestion. Alcohol.

[174] Balbo S., Juanes R.C., Khariwala S., Baker E.J., Daunais J.B., Grant K.A. (2016). Increased levels of the acetaldehyde-derived DNA adductN2-ethyldeoxyguanosine in oral mucosa DNA from Rhesus monkeys exposed to alcohol. Mutagenesis.

